# Enhancing cDC1-mediated anti-tumor immunity limits tumor progression and potentiates anti-PD-1 therapy in intrahepatic cholangiocarcinoma

**DOI:** 10.3389/fimmu.2025.1708962

**Published:** 2025-12-12

**Authors:** Dong-Ni Pei, Yang Song, Ying-Xia Zhou, Bo Shu, Shao-wei Huang, Fa-Zhao Li, Wei-Dong Dai, Bao-Ye Sun

**Affiliations:** 1 Department of General Surgery, the Second Xiangya Hospital of Central South University, Changsha, Hunan, China; 2 Clinical Immunology Research Center, the Second Xiangya Hospital of Central South University, Changsha, Hunan, China; 3 Department of Surgical Operation, the Second Xiangya Hospital of Central South University, Changsha, Hunan, China

**Keywords:** intrahepatic cholangiocarcinoma, tumor microenvironment, immune evasion, anti-PD-1 therapy, conventional dendritic cells

## Abstract

**Purpose:**

Conventional type 1 dendritic cells (cDC1s) mastermind anti-cancer immunity and play a pivotal role in determining the efficacy of cancer immunotherapies. In this study, we sought to decipher the dynamic changes in the tumor immune landscape during intrahepatic cholangiocarcinoma (iCCA) development and harness the therapeutic potential of targeting cDC1s for cancer therapy.

**Methods:**

We constructed spontaneous murine iCCAs via hydrodynamic tail vein injection (HDTVi) of plasmids encoding AKT/YAP. To characterize tumor-infiltrating immune cell populations throughout iCCA carcinogenesis and progression, we performed time-of-flight mass cytometry (CyTOF). To expand and activate cDC1s, we combined Flt3L with poly I:C (FL-pIC) therapy and assessed its therapeutic efficacy in both AKT/YAP-induced iCCAs and a subcutaneous tumor injection model. Flow cytometric analyses were used to evaluate intra-tumoral infiltration levels of cDCs and CD8^+^ T cells.

**Results:**

CyTOF analysis revealed the progressive formation of an immunosuppressive tumor microenvironment as iCCA advances. Crucially, infiltration of cDC1s dramatically decreases in advanced iCCAs compared to early-stage tumors. Combined FL-pIC therapy preferentially expanded CD103^+^ cDC1s, powerfully inhibiting tumorigenesis in AKT/YAP-driven murine iCCAs and sensitizing these tumors to anti-PD-1 therapy. Moreover, FL-pIC therapy markedly suppressed the growth of established mIC-23 subcutaneous tumors.

**Conclusions:**

Our findings demonstrate that recruiting and activating intra-tumoral cDC1s is feasible and essential for driving anti-tumor CD8^+^ T cell immune responses and enhancing anti-PD-1 therapeutic effectiveness in iCCA.

## 1 Introduction

Intrahepatic cholangiocarcinoma (iCCA) ranks the second most common primary liver tumor and accounts for 10-15% of primary liver cancer (1), with continuously increasing mortality and incidence (2, 3). Surgical resection is considered the only potentially curative treatment for iCCA, while only 20%-30% of patients are eligible for resection (4). For the 70%-80% of patients with locally unresectable or distant metastatic disease, systemic therapy such as durvalumab (anti-PD-L1 antibody) combined with standard gemcitabine-cisplatin chemotherapy showed robust and sustained overall survival benefit, but median survival remains limited to approximately 1 year (5). Therefore, it is urgent to identify novel targets and explore more effective anti-cancer therapies for iCCA.

Tumor immune microenvironment (TIME) is a heterogeneous and dynamically reprogramming ecosystem (6). The composition of the heterogeneous TIME is intricate, encompassing a diverse array of lymphocytes and immunosuppressive myeloid subsets. Mounting evidence indicates that the TIME exerts a pivotal influence on anti-cancer immunity and dictates treatment responses to immunotherapy, while the dynamic alterations in these immune cell populations during iCCA initiation and progression remain poorly characterized. Rigorously dissecting the TIME landscape and investigating its therapeutic relevance could profoundly advance our comprehension of the mechanisms underlying tumor immune escape in iCCA.

As the orchestrators of anti-cancer immunity, conventional dendritic cells (cDCs) are a heterogeneous population of antigen-presenting cells that regulate adaptive T-cell immunity against cancer (7). cDCs promote the activation of potent anti-tumor immune responses via numerous mechanisms, including presenting tumor-specific antigens to sustain T cell-mediated tumor elimination. cDCs originate from hematopoietic stem cells (HSCs) in bone marrow and can be further divided into two functionally different subsets, including conventional type 1 DCs (cDC1s) and conventional type 2 DCs (cDC2s) (8). cDC1s specialize in acquiring antigens from tumor cells and cross-presenting these tumor antigens to prime and activate CD8^+^ T cells, whereas cDC2s are more adept at supporting CD4^+^ T cell response (8, 9). Through their high expression of co-stimulatory molecules and potent cytokines like IL-12, cDC1s foster robust T cell activation, vigorous proliferation, and enhanced cytotoxicity. Furthermore, they orchestrate T cell infiltration into the tumor microenvironment and actively support the development of long-lasting immunological memory. Their unique ability to cross-present exogenous antigens on MHC class I molecules proves indispensable in combating tumors and enabling potent responses to immunotherapies (9, 10). However, it remains enigmatic how iCCA cells evade cDC1-mediated immunosurveillance within the TIME and subsequently metastasize to distant organs.

Here, we utilized an AKT/YAP-induced spontaneous murine iCCA model (11, 12) to examine the dynamic TIME landscape during iCCA development. We observed decreased infiltration of cDC1s and CD8^+^ T cells in advanced iCCAs compared with early iCCAs. Moreover, expansion and activation of cDC1s at the tumor site inhibits iCCA carcinogenesis and progression, and enhances tumor responses to anti-PD-1 therapy in murine iCCAs. Our data demonstrate that therapeutic strategies aiming to enhance the functionality of intra-tumoral cDC1s hold great potential to promote effective cancer immune control.

## 2 Materials and methods

### 2.1 Patient cohorts enrolled

This study included 3 iCCA cohorts. (1) scPLC Cohort (13) included scRNA-seq samples from 25 treatment-naive iCCA patients. (2) For SC-iCCA Cohort (14), we reanalyzed scRNA-seq data of 14 iCCA samples. (3) The Bulk RNA-seq data from FU-iCCA cohort were analyzed as previously described (15, 16). The clinical stage of iCCA patients was evaluated according to the American Joint Committee on Cancer (AJCC) 8th edition.

### 2.2 Mice

A total of sixty-seven six-week-old C57BL/6 mice were acquired from Shanghai Ji-hui Co., Ltd. All animals were maintained under specific pathogen-free conditions. Research involving these mice was conducted strictly in accordance with protocols approved by the Institutional Animal Care and Use Committee of the Second Xiangya Hospital of Central South University (Approval No. 20240625).

### 2.3 AKT/YAP-induced murine iCCAs

To establish AKT/YAP-induced spontaneous iCCAs (11, 12), for each C57BL/6 mouse, 20μg pT3-myr-AKT-HA, 20μg pT3-EF1a-FLAG-YAPS127A, and 10μg pCMV-CAT-T7-SB100x dissolved in 2mL Ringer solution were injected through tail vein within 5 seconds.

### 2.4 CyTOF data analysis

Time-of-flight mass cytometry (CyTOF) data analysis was performed by PLTTech Inc (Hangzhou, China) as previously described (17). Live and single CD45^+^ immune cells were gated for subsequent normalization, dimension reduction, and celltype annotation.

### 2.5 Subcutaneous tumor model

Ten six-week-old female C57BL/6 mice were injected subcutaneously in the left flank with 5×10^6^ mIC-23 cells suspended in 100μL PBS. Three weeks after cell implantation, mice were randomized into indicated groups prior to treatment. Tumor growth was measured by caliper every 3–4 days and tumor volume was calculated using the formula length (mm) × width^2^ (mm)/2. On the 35th day after tumor cell implantation, mice were euthanized by intraperitoneal injection of sodium pentobarbital at a lethal dose of 150 mg/kg body weight and tumors were harvested for further analyses.

### 2.6 Treatments

For expansion and activation of cDC1s, we combined Flt3L with poly(I:C) therapy as previously reported (9, 18). C57BL/6 mice were injected intraperitoneally with 30μg active recombinant mouse Flt3L protein (RP01058, ABclonal Technology) dissolved in 100μL PBS or control PBS for 9 consecutive days. High molecular weight poly(I:C) (InvivoGen) was injected intraperitoneally for AKT/YAP murine iCCAs (200μg/dose in 100μl PBS), or intratumorally (50μg/dose in 30μl PBS) for mIC-23 subcutaneous tumor injection models on day 5 and 9 after Flt3L administration. For anti-PD-1 combination therapy, mice (n=5/group) were treated intraperitoneally every 3 days with 10 mg/kg anti-PD-1 monoclonal antibody (BioXCell, BE0146) or isotype control antibody (BioXCell, BE0089). Mice were monitored routinely to examine tumor formation and treatment tolerability, including (1) Tumor size ≤2000 mm³for subcutaneous tumors, (2) Body weight loss threshold <20%, and (3) Daily monitoring for distress signs (e.g., lethargy, poor grooming). At the endpoint, serum was collected from mice from each group (n=5/group) and analyzed for liver function tests (ALT and AST) and cytokine TNF-α levels (Servicebio^®^Mouse TNF-α MPCLIA Kit, Cat# GLM0004) to detect any treatment-associated hepatic toxicity or systemic proinflammatory effects. Mice were sacrificed by intraperitoneal injection of sodium pentobarbital (150 mg/kg body weight) and tissues were harvested for further analyses.

### 2.7 *In vivo* imaging

We applied *in vivo* bioluminescence imaging to monitor tumor formation. Mice were anesthetized by inhaling an isoflurane-oxygen mixed gas, with the isoflurane concentration maintained at 2%-2.5% and then injected intraperitoneally with 150 mg/kg D-luciferin (D115509, aladdin). 10 minutes later, *in vivo* imaging was performed using an IVIS Spectrum system (Perkin Elmer). Luciferase signal was quantified as average Radiance [Photons/second/cm^2^/sr] of region of interest using Living Image software 4.4 (Perkin Elmer).

### 2.8 Cell lines

Mouse iCCA cell line mIC-23 cells were established as described before (19) and cultured in DMEM containing 10% FBS in a 37 °C humidified incubator with 5% CO_2_.

### 2.9 Flow cytometry

Flow cytometric analyses were performed using BD FACSAria III. Data were analyzed using FlowJo (BD). Zombie NIR Fixable Viability Kit (BioLegend, Cat#423105) was used to exclude dead cells, and anti-CD16/CD32 antibody (BioLegend, Cat#156603) was used to block non-specific binding of antibodies via Fc receptors. The following antibodies used for flow cytometry were purchased from BioLegend: anti-CD45-FITC (30-F11), anti-CD11c-BV421 (N418), anti-CD103-PE (2E7), anti-CD11b-APC (M1/70), anti-MHC II-I-A/I-E-PE-Cy7 (M5/114.15.2), anti-CD8a-PE-Cy7 (53-6.7), anti-Granzyme B-PE (QA16A02). Intracellular staining of Granzyme B was done using the Cyto-Fast™ Fix/Perm Buffer Set (Cat#426803) from BioLegend. After gating on live and single cells, the following immune cell populations were identified: DCs (CD45^+^CD11c^+^ MHCII^+^), CD103^+^cDC1s (CD45^+^CD11c^+^MHCII^+^CD103^+^CD11b^-^), CD11b^+^cDC2s (CD45^+^CD11c^+^MHCII^+^CD103^-^CD11b^+^), cytotoxic CD8^+^ T cells (CD45^+^CD8^+^GZMB^+^).

### 2.10 Immunohistochemistry (IHC) and immunofluorescence (IF) staining

IHC and IF staining was performed according to the procedures detailed before (20). The antibodies used for IHC staining were anti-CK19 (1:1000, Cat# ab52625, Abcam), anti-CD8A (1:2000, Cat# ab209775, Abcam) and PD-1 (1:1000, Cat# ab214421, Abcam).

### 2.11 RT-qPCR

Total RNA was extracted using TRIzol (Invitrogen). Subsequently, reverse transcription was meticulously conducted with PrimeScript RT Master Mix (Takara, Japan, RR036A). qPCR analysis was then executed on a Real-Time PCR system (Applied Biosystems, USA), leveraging the TB-Green-based PCR kit (Takara, Japan). β-actin served as the internal control. The relative quantification (2^-ΔΔCt^) method was used to analyze the fold-changes of mRNA expression levels relative to a control sample.

### 2.12 Statistical Analysis

Statistical analyses were performed using R package (v4.1.2) and Graphpad (v9.0) software. Student’s t-test or Mann-Whitney test was used to compare the differences of quantitative variables between two groups. Two-tailed P < 0.05 was considered as statistically significant.

## 3 Results

### 3.1 High-dimensional analysis of the TIME landscape in early and advanced iCCAs

**Figure 1:**
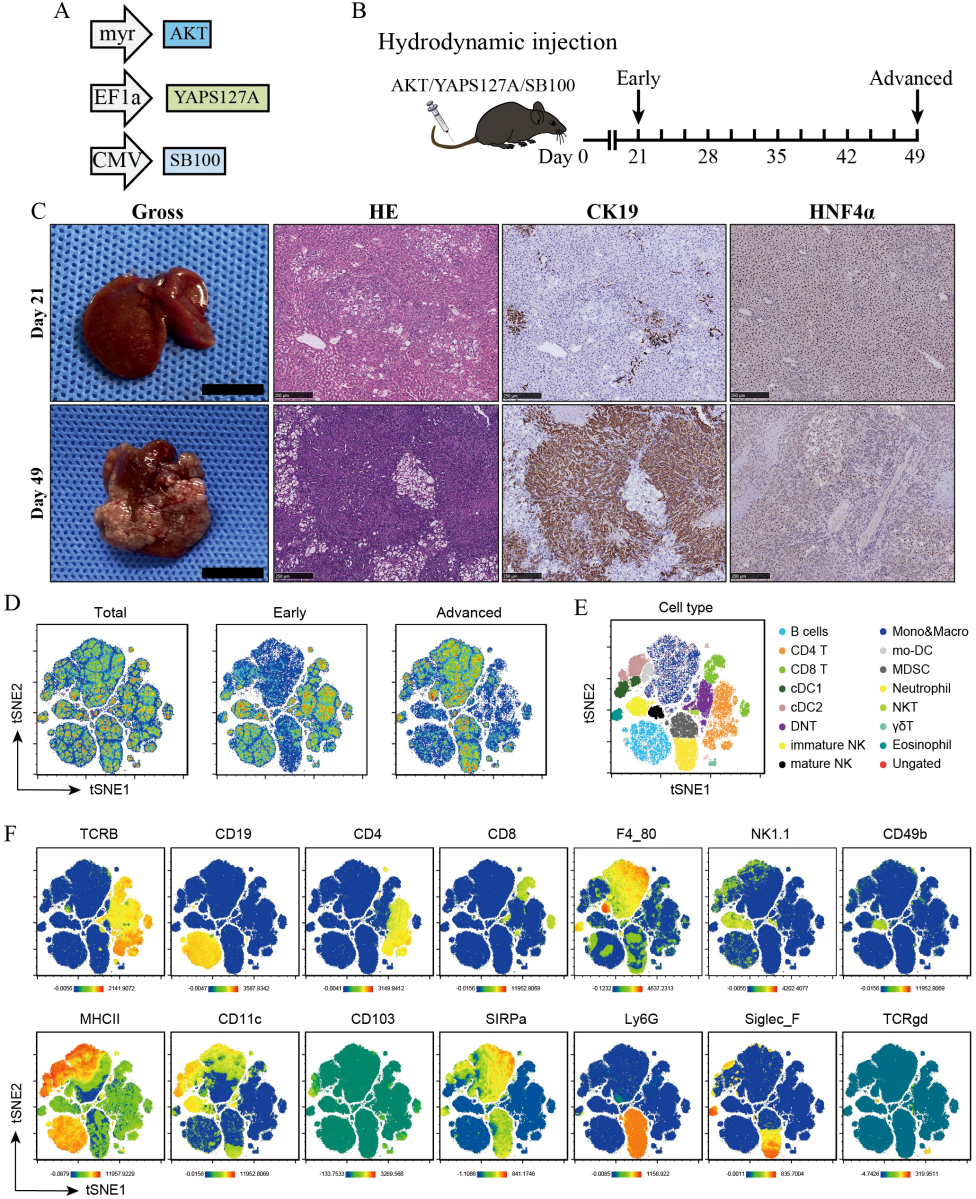
High-dimensional analysis of the tumor-infiltrating immune cell populations in early and advanced iCCAs. **(A)** Schematic of vectors overexpressing AKT, YAPS127A, and SB100 injected into mice. **(B)** Mouse liver tissues were collected on day 21 and 49 representing early and advanced stages of iCCA after hydrodynamic injection of indicated plasmids. **(C)** Gross images, H&E staining, IHC staining of CK19 and HNF4α of liver tumors from indicated time points. **(D)** t-SNE plots showing tumor-infiltrating CD45^+^ immune cells in AKT/YAP murine iCCAs from indicated groups. **(E)** t-SNE plots of tumor-infiltrating B cells, CD4^+^ T, CD8^+^ T, cDC1s, cDC2s, Double-negative T cells, Immature NK cells, Mature NK cells, Monocytes & macrophages, Monocyte-derived dendritic cells (mo-DC), Myeloid-derived suppressor cells (MDSCs), Neutrophils, NKT cells, γδT cells, and Eosinophils identified by CyTOF analysis. **(F)** t-SNE plots showing relative expression of corresponding lineage markers of tumor-infiltrating immune cells.

Previous studies have demonstrated that aberrant activation of Yes-associated protein (YAP) along with AKT induces iCCA carcinogenesis in mice (11, 12). In our study, ectopic oncogene expression into mouse liver was accomplished by hydrodynamic tail vein injection of the Sleeping Beauty (SB100) transposon-mediated transfection system with transduction of constitutively active AKT (myr-AKT) and human YAP (huYAP), YAPS127A (**Figures 1A, B**). The mice were shown to have microscopic tumor lesions as early as 3 weeks which were confirmed to be iCCA based on histological analysis and IHC staining of CK19 (**Figure 1C**). On day 49, large macroscopic iCCA tumor nodules were spread all over the liver surface, indicating an advanced stage of iCCA. Therefore, we collected mouse iCCA tissues on days 21 and 49 representing early and advanced stages of iCCA, respectively.

To thoroughly characterize the dynamic immune landscape during iCCA development, we used time-of-flight mass cytometry (CyTOF) and analyzed AKT/YAP-induced iCCAs at two different time points (**Figure 1D**). With this approach, CD45^+^ immune cells within TME were gated and divided into lymphoid and myeloid compartments (**Supplementary Figure S1A** and **Figure 1D**). We observed an accumulation of myeloid compartments that paralleled a reduced tumor-infiltrating lymphocyte (TIL) density over time, suggesting that the expansion of immunosuppressive myeloid cells could impair anti-tumor immunity in iCCAs (**Figures 1D, E**). The CyTOF panel enabled us to further identify the specific immune cell populations according to the expression of corresponding lineage markers, including B cells, CD4^+^ T cells, CD8^+^ T cells, cDC1s, cDC2s, Double-negative T cells (DNT), Immature NK cells, Mature NK cells, Monocytes & macrophages, Monocyte-derived dendritic cells (mo-DC), Myeloid-derived suppressor cells (MDSCs), Neutrophils, NKT cells, γδT cells, and Eosinophils (**Figures 1E, F**).

### 3.2 The infiltration of cDC1s is decreased in advanced iCCAs compared with early iCCAs

**Figure 2:**
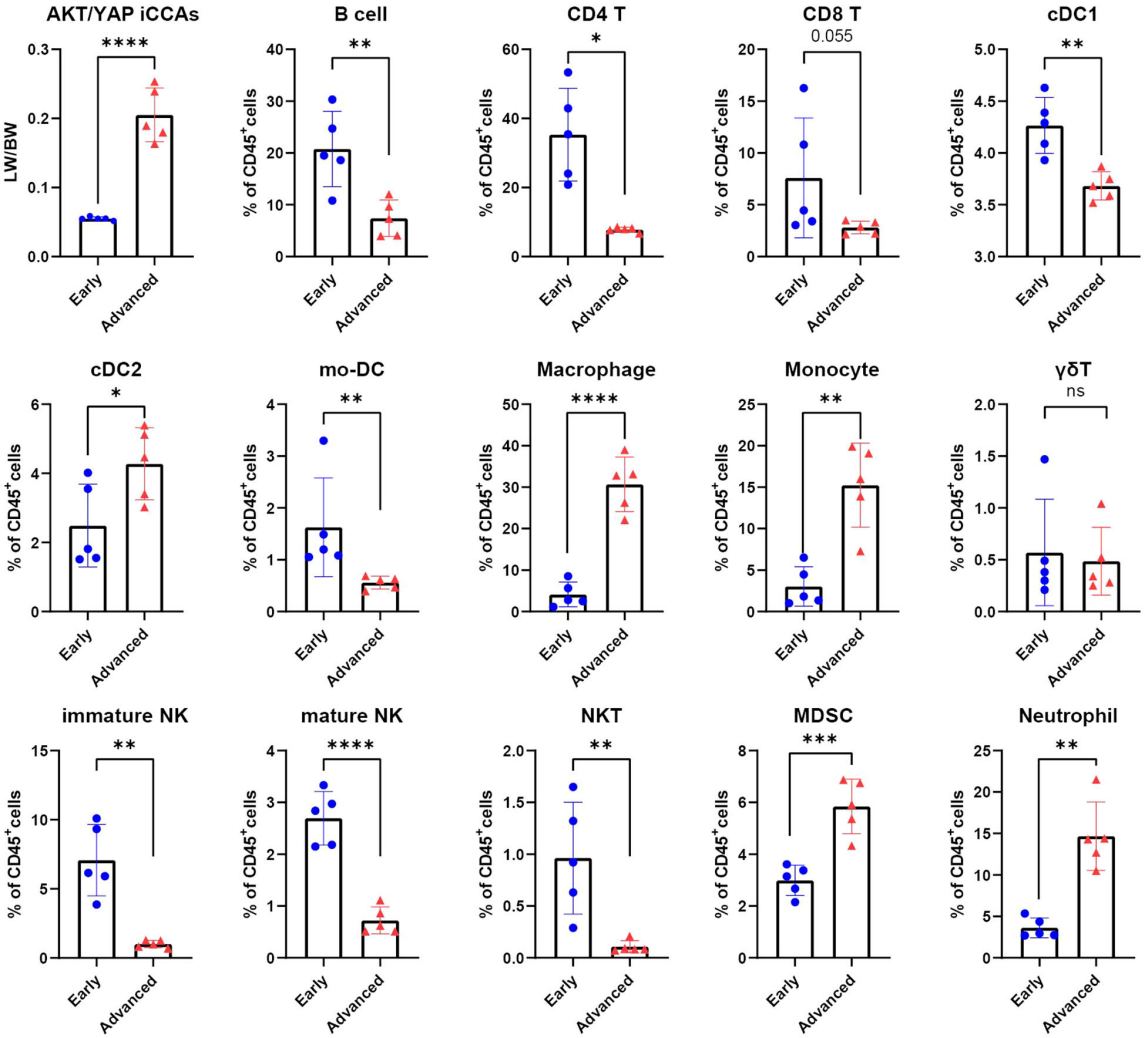
The infiltration of cDC1s is decreased in advanced iCCAs compared with early iCCAs. Tumor burden evaluated by liver weight versus body weight (LW/BW) ratios and tumor-infiltrating levels of B cells, CD4^+^ T, CD8^+^ T, cDC1s, cDC2s, mo-DC, macrophages, monocytes, γδT cells, Immature NK cells, Mature NK cells, NKT cells, MDSCs, and Neutrophils from early and advanced iCCAs assessed by CyTOF. ns, not significant; *p < 0.05, **P < 0.01, ***p < 0.001, ****p < 0.0001.

We then calculated the percentages of these immune cell subsets in each individual sample. Consistently, tumor-infiltrating lymphoid subsets such as B cells, CD4^+^ T cells, CD8^+^ T cells, immature NK cells, mature NK cells, and NKT cells were significantly reduced in advanced iCCA tumors. In contrast, myeloid subsets like Monocytes, Macrophages, MDSCs, and Neutrophils were significantly increased in the TME during iCCA progression (**Figure 2**). Intriguingly, the proportions of cDC1s and cDC2s displayed opposite trends during iCCA initiation and progression. Intra-tumoral cDC1s were significantly decreased, whereas cDC2s were increased in late-stage iCCAs compared with early-stage iCCAs (**Figure 2**). cDC1s are crucial for anti-cancer immunity, and their abundance within tumors is associated with CD8^+^ T cell-mediated immune rejection, favorable overall patient survival, and better clinical response to immunotherapy (21, 22). A critical anti-tumor function of cDC1s involves cross-priming and reviving tumor-specific CD8^+^ T cells. As we observed that cDC1s and CD8^+^ T cells were excluded from TME as iCCAs continue to progress, we postulated that shrinkage of cDC1s might correlate with advancing disease and promote tumor immune evasion.

### 3.3 scRNA-seq analyses identify decreased infiltration of cDC1s among advanced iCCA cases

**Figure 3:**
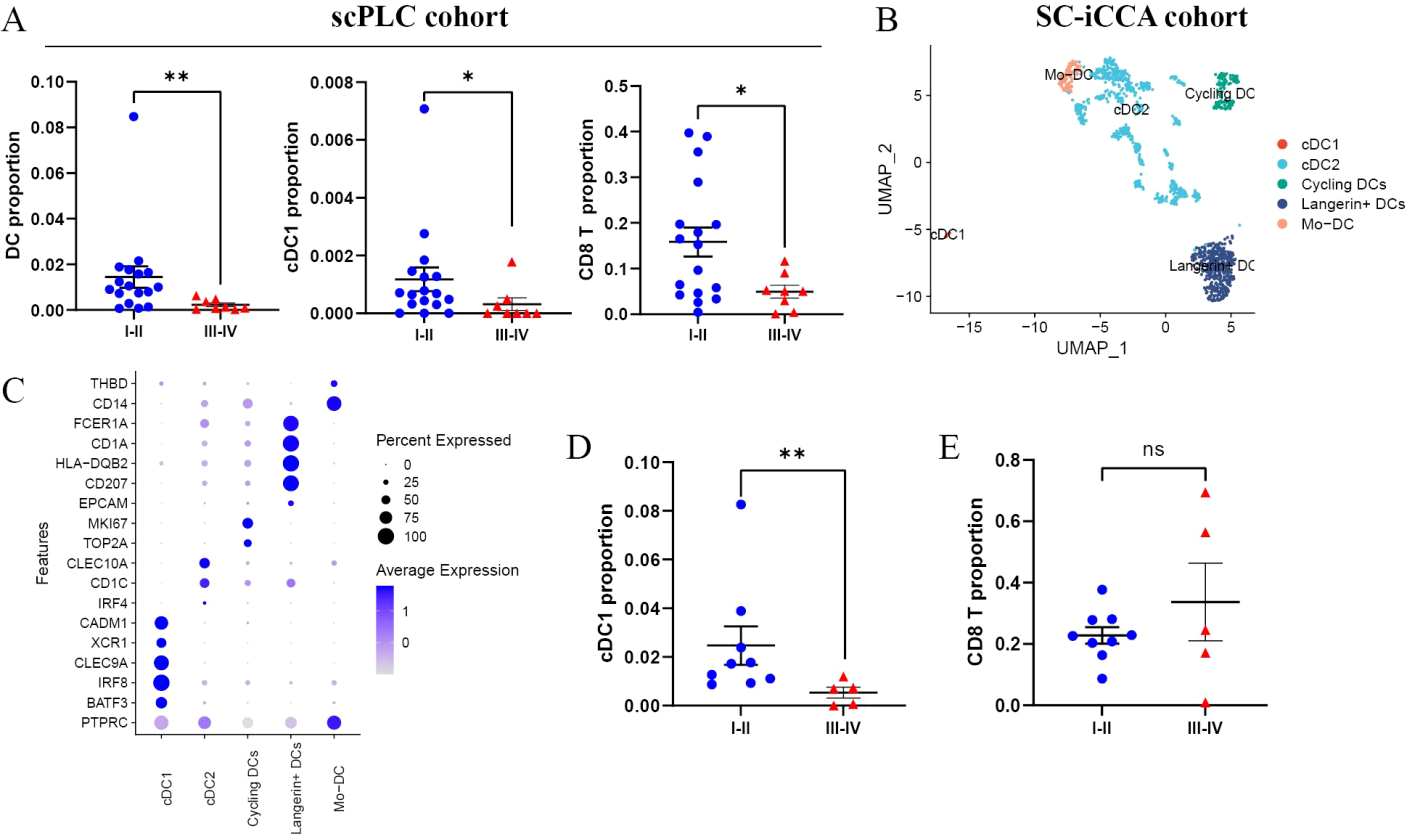
scRNA-seq analyses identify reduced infiltration levels of cDC1s among advanced iCCAs. **(A)** Comparison of the fractions of DCs, cDC1s, and CD8^+^ T cells between iCCA samples with clinical stage III-IV diseases and those with clinical stage I-II disease in scPLC cohort. **(B)** UMAP plots visualizing DC subsets from scRNA-seq profiling of 14 iCCA samples in SC-iCCA cohort. DC subsets are distinguished by colors. **(C)** Dot plot showing the expression pattern of marker genes of indicated DC subsets in SC-iCCA cohort. The fractions of cDC1s **(D)** and CD8^+^ T cells **(E)** among CD45^+^ immune cells in clinical stage III-IV iCCA samples compared with clinical stage I-II iCCAs. ns, not significant; *p < 0.05, **P < 0.01.

To validate the above findings in human iCCA samples, we re-analyzed 2 scRNA-seq datasets of iCCA cohorts from previous studies. In scPLC cohort (13), iCCA patients with clinical stage III-IV diseases showed a significantly lower abundance of DCs, cDC1s, and CD8^+^ T cells than patients with clinical stage I-II disease (**Figure 3A**). In SC-iCCA cohort (14), scRNA-seq analyses identified decreased infiltration of cDC1s among advanced iCCA cases (**Figures 3B-E**), supporting impaired cDC1-mediated immune surveillance as tumors develop and evolve over time. Taken together, these findings suggested that cDC1s are excluded from early tumor stages, which might curb anti-tumor immunity and contribute to iCCA progression.

To explore mechanisms accounting for the impaired cDC1s infiltration into advanced iCCAs, we evaluated the expression levels of several tumor-derived factors (9, 19, 21, 23) known to impact cDC1 recruitment/function by performing qPCR of frozen mouse iCCA tissues collected from early and advanced stages. Intriguingly, we found that expression levels of Ptgs1, Cxcl9, Cxcl10, as well as Ctnnb1 (encoding β-catenin) and β-catenin target genes Axin2 and Ccnd1 were significantly increased, while DC chemokine CXCL12 was down-regulated in advanced iCCA tissues compared with early-stage iCCAs (**Supplementary Figures S2A-D**). This could in part account for the reduced cDC1s in advanced iCCAs.

### 3.4 Enhancing cDC1s infiltration by FL-pIC inhibits iCCA formation and progression and restores anti-tumor CD8^+^ T cell immunity

**Figure 4:**
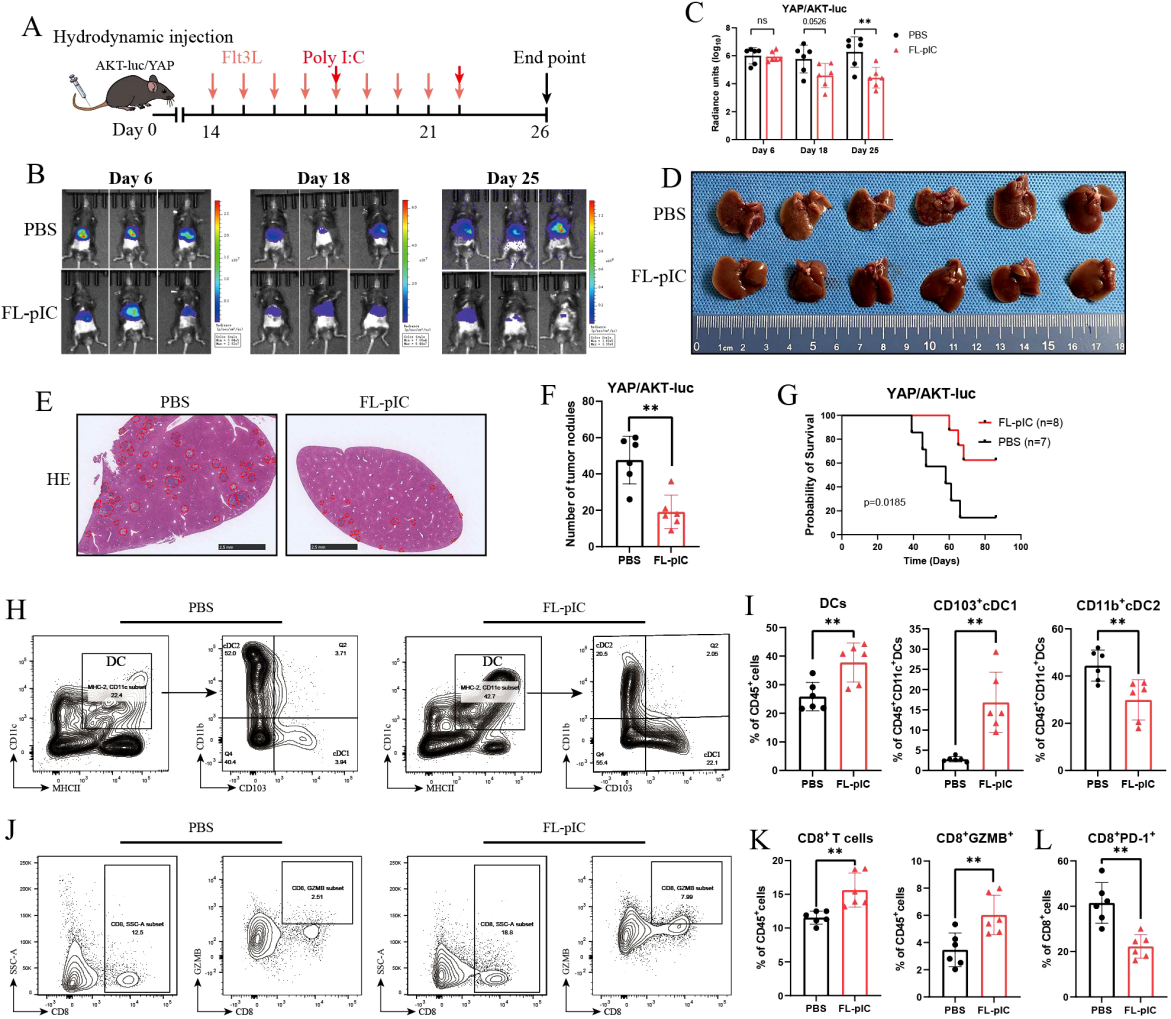
Expansion and activation of cDC1s by combined Flt3L and poly I:C therapy leads to decreased tumor formation and increased survival in murine iCCAs. **(A)** Treatment scheme for 9 daily injections of Flt3L (30μg/dose) followed by poly(I:C) (200 μg/dose or PBS in AKT/YAP-induced spontaneous murine iCCAs. **(B)** Bioluminescence imaging on day 6, 18 and 25 after hydrodynamic injection of indicated plasmids into PBS or FL-pIC-treated mice. **(C)** Quantification of normalized luciferase signal 6, 18, and 25 days after injection of plasmids. **(D)** Gross liver images from PBS and FL-pIC-treated group. **(E)** Representative H&E staining of livers and statistical results of tumor nodules per mouse from indicated groups **(F)**. **(G)** Survival curves of C57BL/6 mice receiving PBS or FL-pIC therapy. **(H)** Representative flow cytometric plots showing CD103 and CD11b expression on CD45^+^ MHCII^+^ CD11c^+^ DCs, and frequency of total DCs, CD103^+^ cDC1 and CD11b^+^ cDC2 in murine iCCAs **(I)**. **(J)** Representative flow cytometric plots showing percentage of CD8^+^ T cells and CD8^+^GZMB^+^ T cells among CD45^+^ cells from indicated groups **(K)**. **(L)** Expression levels of PD-1 on CD8^+^ T cells detected by multiplex immunofluorescence (mIF) staining. ns, not significant; **P < 0.01.

Systemic injection of Fms-like tyrosine kinase 3 ligand (Flt3L) has been reported to induce the expansion of immature cDCs at the tumor sites (9), whereas immature DCs can’t efficiently prime T cells. Therefore, we combined the administration of a toll-like receptor 3 (TLR3) agonist poly(I:C) to promote the maturation and activation of Flt3L-mobilized cDCs (9, 18, 24). We then explored the therapeutic effects of combined FL-pIC on tumor formation using AKT/YAP-induced spontaneous murine iCCA model (**Figure 4A**). Luciferase signal measured by bioluminescence imaging was equivalent in mouse livers between PBS and FL-pIC group six days after hydrodynamic injection of AKT-luc, YAP, and SB100 plasmids, indicating similar injection and hepatocyte transfection efficiency (**Figures 4B, C**). Compared with PBS group, FL-pIC therapy significantly inhibited tumor formation as evidenced by the drastic reduction in luciferase signals and tumor nodules, suggesting effective clearance of luciferase-expressing cancer cells (**Figures 4C-F**). Moreover, the overall survival time of AKT/YAP mice receiving FL-pIC therapy was prolonged compared with that of corresponding mice receiving PBS (**Figure 4G**). Flow cytometric analysis revealed that FL-pIC therapy led to dramatic expansion of cDCs, predominantly CD103^+^ cDC1s rather than CD11b^+^ cDC2s in early iCCA lesions (**Figures 4H, I** and **Supplementary Figure S3A**). Besides, FL-pIC treatment increased the abundance of tumor-infiltrating CD8^+^ T cells and CD8^+^ GZMB^+^ T cells, while reducing CD8^+^ PD-1^+^ T cells in comparison to control group (**Figures 4J-L** and **Supplementary Figures S3B, C**). In summary, these data suggest that FL-pIC therapy enhances cDC1s infiltration into early iCCAs, which could efficiently prime and activate CD8^+^ T cells and attenuates tumor formation.

### 3.5 Enhancing cDC1s infiltration into established iCCAs leads to tumor regression

**Figure 5:**
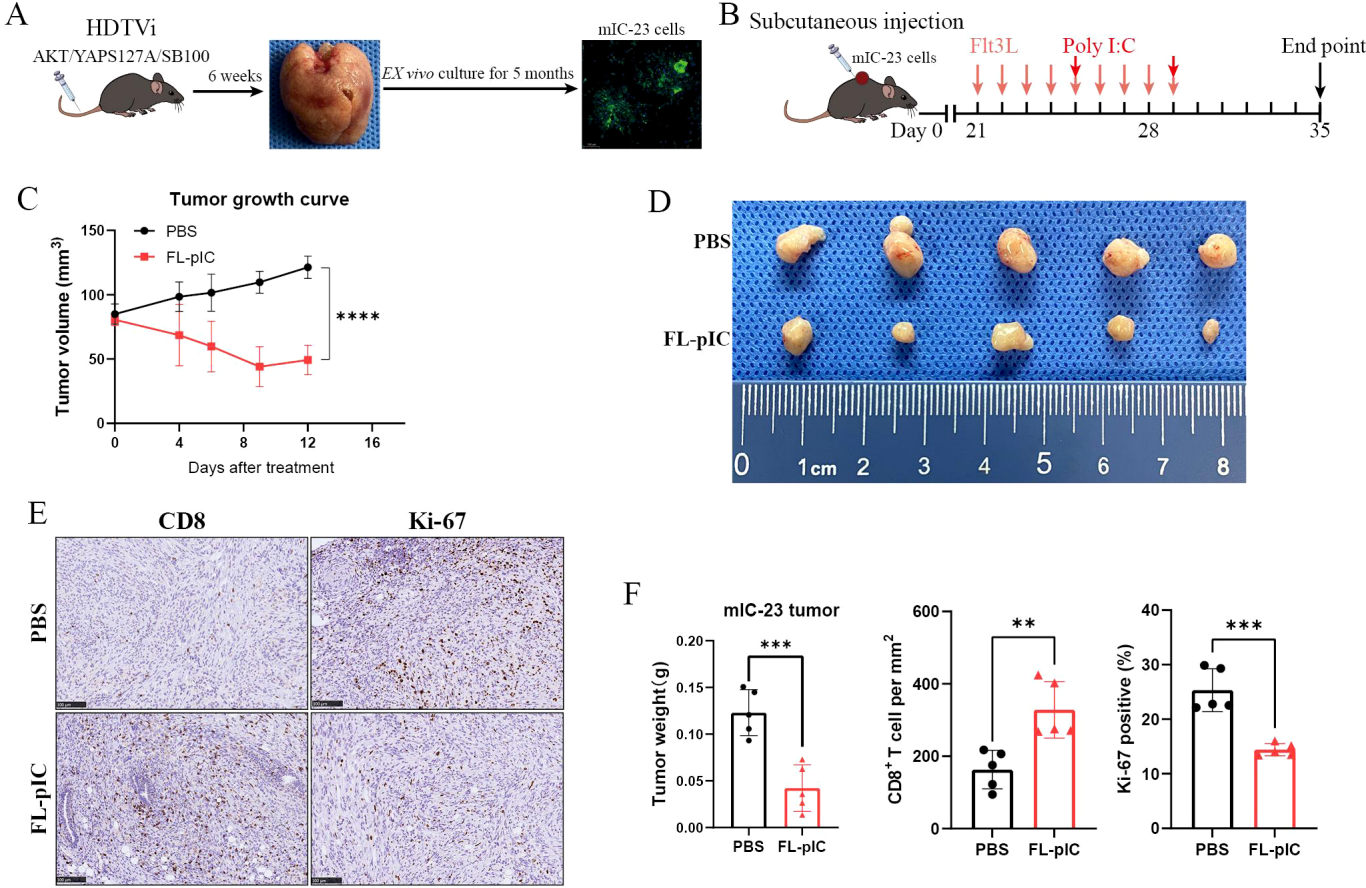
Combined Flt3L and poly I:C therapy inhibits mIC-23 subcutaneous tumor growth. **(A)** A mouse iCCA cell line mIC-23 was generated from AKT/YAP-induced iCCAs after 6 months of *ex vivo* culture. Anti-CK19 antibody was used for IF staining of mIC-23 cells. **(B)** Treatment schedule for 9 daily injections of Flt3L (30μg/dose) followed by intratumoral injection of high molecular weight poly(I:C) (50μg/dose) or PBS in mIC-23 subcutaneous tumor model. **(C)** Growth curves of mIC-23 subcutaneous tumors from indicated groups after treatment initiation. **(D)** Pictures of mIC-23 subcutaneous tumors from indicated groups. **(E)** Representative IHC staining images for CD8 and Ki-67 of mIC-23 tumor tissues. Scale bar: 100μm. **(F)** Statistical results of tumor weight, CD8^+^ T cells, and Ki-67 positive cells from indicated groups. **P < 0.01, ***P < 0.001, ****p < 0.0001.

Next, we examined the effects of FL-pIC therapy on established iCCAs using a subcutaneous tumor injection model. A mouse iCCA cell line mIC-23 was established from AKT/YAP-induced iCCAs as detailed before (19) (**Figure 5A**). FL-pIC therapy significantly inhibited *in vivo* growth of mIC-23 cells (**Figures 5B-D**). Moreover, IHC analysis suggested that FL-pIC treatment promoted intra-tumoral accumulation of CD8^+^ T cells and suppressed tumor cell proliferation in subcutaneous iCCAs (**Figures 5E, F**).

### 3.6 FL-pIC therapy enhances tumor responses to anti-PD-1 immunotherapy in murine iCCAs

**Figure 6:**
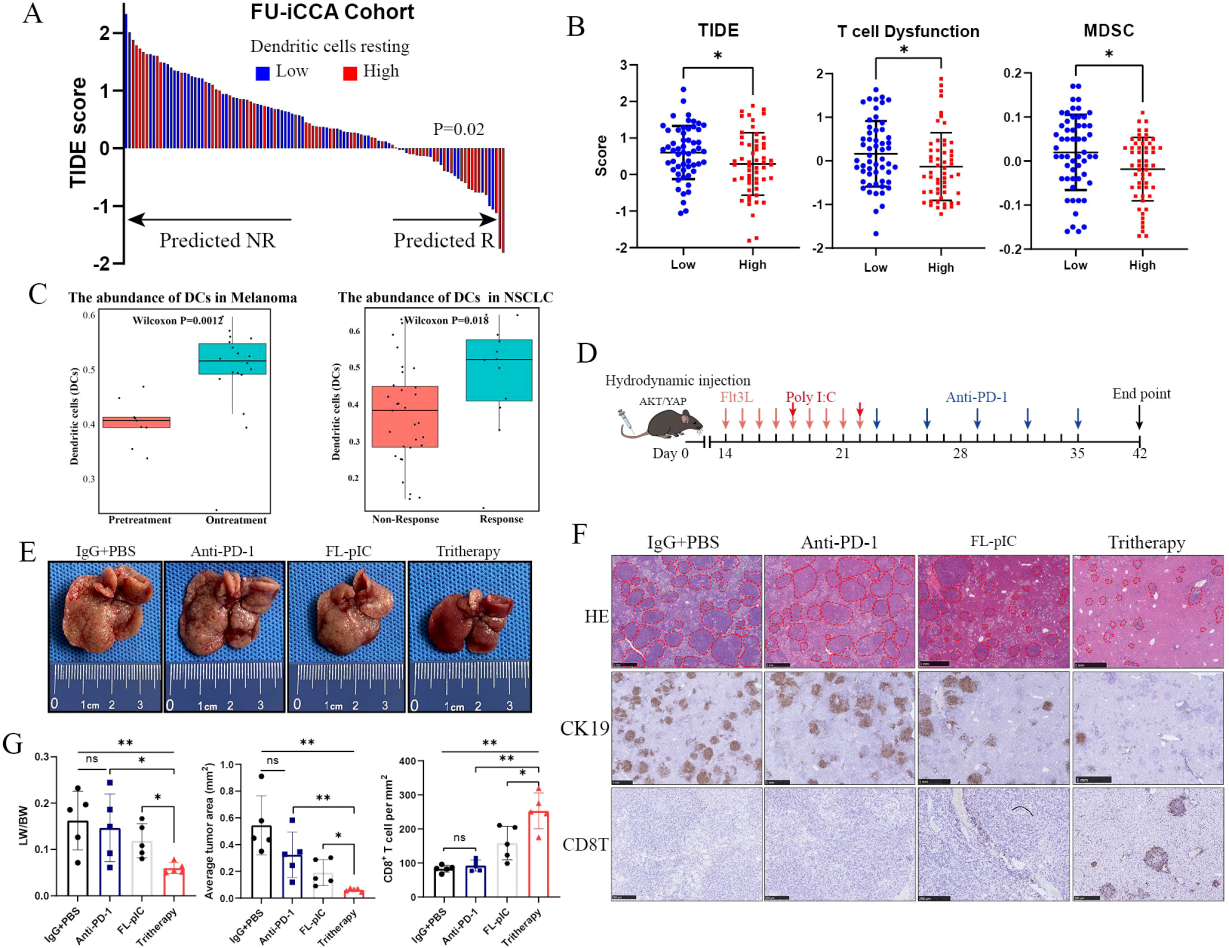
Combined Flt3L and poly I:C therapy enhances tumor responses to PD-1 blockade in murine iCCAs. **(A)** Prediction of ICB therapy response in patients with high versus low infiltration of tumor-resting DCs in FU-iCCA cohort using the TIDE signature. **(B)** Analysis of TIDE, T cell dysfunction and MDSC signature scores by TIDE algorithm in patients with high or low infiltration of DCs. **(C)** The abundance of DCs between Pre-treatment and On-treatment (Pre vs on) samples in melanoma (left) and Responsive versus Non-Responsive (R vs NR) samples in NSCLC (right). **(D)** Schematic representation of the treatment schedule for systemic administration of Flt3L (30μg/dose) followed by poly(I:C) (200μg/dose), anti-PD-1 (10mg/kg), or combination therapy. **(E)** Representative liver images from AKT/YAP iCCA models that received indicated treatments (5 mice per group). **(F)** H&E staining, CK19, and CD8 staining images of liver tumors from indicated groups. **(G)** Tumor burden evaluated by liver weight versus body weight ratios (LW/BW), average area of tumor nodules, and quantification of tumor-infiltrating CD8^+^ T cells from indicated groups assessed by IHC analysis (cells/mm^2^). ICB, immune checkpoint blockade. MDSC, myeloid-derived suppressor cells. TIDE, Tumor Immune Dysfunction and Exclusion. NSCLC, non-small cell lung cancer. ns, not significant; *p < 0.05, **P < 0.01.

iCCA is notorious for its poor response to immune checkpoint blockades (ICBs), including anti-PD-1/PD-L1 therapy. Based on RNA-seq data from FU-iCCA cohort, we used Tumor Immune Dysfunction and Exclusion (TIDE), an algorithm integrating gene signatures of T cell dysfunction and exclusion to predict ICB response (25).TIDE analysis revealed that patients with high DC infiltration was associated with better ICB response according to their lower TIDE, T cell dysfunction and MDSC signature scores when compared with those with low DC infiltration (**Figures 6A, B**). Furthermore, public data analysis showed that DCs expanded in melanoma samples receiving anti-PD-1 therapy compared with pre-treatment samples (26). Besides, samples with high abundance of DCs had significantly better responses to anti-PD-1 therapy in non-small cell lung cancer (27) (**Figure 6C**).

Accordingly, we speculated that the scarcity of intra-tumoral cDC1s limited the activation of CD8^+^ T cells and thus impaired anti-PD-1 efficacy in iCCAs. Therefore, we utilized AKT/YAP-induced iCCA model to examine whether FL-pIC therapy sensitizes iCCAs to anti-PD-1 therapy (**Figure 6D**). Mice treated with IgG control, anti-PD-1, FL-pIC, or FL-pIC/anti-PD-1 did not display any significant differences in body weight during the treatment (**Supplementary Figure S4A**) and normal alanine aminotransferase, aspartate aminotransferase and inflammatory cytokine TNF-α levels were observed (**Supplementary Figures S4B–D**). These results indicate that this treatment regimen seems to be tolerated and does not appear to cause significant hepatotoxicity in mice. As expected, anti-PD-1 monotherapy failed to inhibit iCCA formation and progression (**Figure 6E**). Meanwhile, PD-1 blockade combined with FL-pIC administration resulted in a significant reduction of tumor burden, accompanied by robust infiltration of CD8^+^ T cells into tumors (**Figures 6E–G** and **Supplementary Figure S4E**). These findings suggest that enhanced infiltration of cDC1s by FL-pIC therapy could limit iCCA progression and increase tumor response to anti-PD-1 therapy.

## 4 Discussion

Cancer prognosis and therapy are being re-defined on two complementary fronts. Clinically, the cachexia index (CXI) has emerged as a powerful predictor of poor outcome (28), while the BILCAP trial has established adjuvant capecitabine as the standard of care after resection of biliary-tract cancer (29). Biologically, iCCAs are fuelled by a background of chronic, low-grade inflammation that overlaps with obesity, type 2 diabetes and metabolic syndrome (30). Genomically, IDH1/2 and FGFR alterations delineate distinct iCCA subsets whose microenvironment can be reprogrammed by targeted agents and immunotherapy (31, 32). In this study, using high-dimensional CyTOF analysis of AKT/YAP-induced spontaneous murine iCCAs from both early and late stages, we observed that cDC1s and CD8^+^ T cells are progressively excluded from the tumor microenvironment beginning at early tumor stages during iCCA development. This exclusion was also evident for other lymphoid subsets such as B cells and NK cells, suggesting broad immune evasion mechanisms. Further supporting these findings, scRNA-seq analysis of two independent human iCCA cohorts consistently identified significantly reduced infiltration of cDC1s in patients with advanced-stage iCCAs, highlighting the clinical relevance of our observations. The paucity of tumor-infiltrating cDC1s could in turn impair the priming and activation of tumor-specific CD8^+^ T cells, leading to defective cytotoxic responses and ultimately contributing to immune tolerance and cancer progression. Thus, cDC1s represent crucial cellular targets for novel therapeutic interventions in iCCA. Strategies aimed at potentiating the anti-tumor functions of cDC1s—such as enhancing their recruitment, antigen presentation capacity, or cross-talk with T cells—could elicit robust short-term and long-term anti-cancer immunity, offering immense therapeutic value for aggressive malignancy.

As the most potent antigen-presenting cells for priming cancer-specific CD8^+^ T cells, cDC1s play a crucial role in efficiently launching anti-tumor immunity. These specialized cells excel at capturing, processing, and presenting tumor-associated antigens to naïve T cells, thereby sparking a precisely targeted and adaptive immune response (9, 10). However, cDC1s are sparsely distributed within the tumour microenvironment and often functionally hijacked by tumour-derived immunosuppressive factors such as prostaglandin E2 (PGE2) and granulocyte colony-stimulating factor (GCSF). These factors impair the ability of cDC1s to effectively present tumour antigens and prime antitumour T-cell responses, thereby facilitating immune evasion. PGE2, for instance, modulates dendritic cell differentiation and suppresses their maturation, while GCSF alters haematopoietic development favoring myeloid-derived suppressor cells over functional cDC1s. This subversion of cDC1 function represents a key mechanism by which tumours dampen antitumour immunity and undermine the efficacy of immunotherapies (9, 21, 23).We have also previously reported that β-catenin-mediated suppression of CXCL12 expression serves as a key mechanism accounting for the defective DC recruitment and lymph node metastasis in iCCA. This impairment in DC recruitment subsequently weakens the antitumor immune response and facilitates the dissemination of cancer cells to regional lymph nodes (19).

Systemic administration of Flt3L, followed by a TLR3 agonist poly(I:C) or CD40 agonist, has been reported to successfully promote the expansion and activation of tumor-residing cDC1s and enhances tumor responses to anti-PD-L1 therapy (9, 18). *In situ* vaccination (ISV) combining Flt3L, local radiotherapy, and a TLR3 agonist, led to systemic clinical cancer remission and potentiation of PD-1 blockade in patients with advanced stage indolent non-Hodgkin’s lymphomas (iNHLs) (33). CD40 agonist-mediated activation of macrophages and DCs also enhances response to anti-PD-1 therapy in multiple murine iCCA models (12). Using an AKT/YAP-induced spontaneous murine iCCA model (11, 12), we examined the effects of combined FL-pIC therapy on iCCA tumorigenesis and progression. Consistent with previous findings, combined FL-pIC therapy preferentially induced the recruitment of CD103^+^ cDC1s rather than CD11b^+^ cDC2s, as well as cytotoxic CD8^+^GZMB^+^ T cells. Despite great success in cancer immunotherapy, ICBs like anti-PD-L1/PD-1 therapy alone showed limited efficacy in the treatment of advanced iCCAs (34, 35), highlighting the necessity of combing other therapeutic options. cDC1s activities are key determinants of the efficacy of immunotherapies, including anti-PD-1 therapy. Here, we found that FL-pIC therapy rendered murine iCCAs responsive to anti-PD-1 therapy. PD-1 blockade combined with FL-pIC resulted in a significant inhibition of tumor progression. Our findings substantiate that expanding and activating intra-tumoral cDC1s is feasible and critical for effective tumor immune control.

Several limitations of this study should be noted. First, while we explored the inherent potential of targeting cDC1s for cancer therapy, the majority of our findings were derived from preclinical mouse models of iCCA. Although these models provide valuable mechanistic insights, they may not fully recapitulate the complexity and heterogeneity of human iCCA. A patient-derived tumor xenograft (PDX) model or humanized system treated with FL-pIC would dramatically enhance its translational relevance. Moreover, clinical evaluation of the safety, tolerability, and efficacy of combined treatment with FL-pIC and anti-PD-L1/PD-1 therapy following standard gemcitabine-cisplatin chemotherapy will be essential to validate our results and provide more solid evidence for potential translational applications. Besides, without cDC1-specific loss-of-function experiments, our current data provide correlative evidence and functional gain but lack conclusive demonstration of necessity. Additionally, the molecular mechanisms underlying the impaired infiltration and function of cDC1s in iCCA remain incompletely understood. Further investigation is warranted to elucidate the signaling pathways, tumor microenvironment interactions, and possible immunosuppressive factors that contribute to the dysfunction of cDC1s. Such studies could identify novel targets for enhancing cDC1 recruitment and activation, thereby improving the efficacy of combination immunotherapies in iCCA patients. Tumor-derived immunosuppressive factors like β-catenin activation, PGE2, GCSF, impaired intra-tumoral cDC1 chemokine production like CCL4, CCL5, CXCL12, or defects in cDC1 precursor differentiation in the bone marrow mediating intra-tumoral cDC1 dysfunction should also be further explored and validated (9, 19, 21, 23).

In conclusion, our findings demonstrate that the expansion and functional activation of cDC1s significantly enhances anti-tumor immunity and represents a promising therapeutic strategy for restricting the carcinogenesis and progression of iCCA. Moreover, targeting cDC1s may synergize with existing immunotherapies, potentially overcoming resistance mechanisms and improving clinical response rates in iCCA patients. Further investigation into the molecular pathways regulating cDC1 recruitment and activation could provide novel insights for combination treatment approaches aimed at achieving durable anti-tumor effects.

## Data Availability

The original contributions presented in the study are included in the article/**Supplementary Material**. Further inquiries can be directed to the corresponding authors.
