# Supplementary material for: Enhancing cDC1-mediated anti-tumor immunity limits tumor progression and potentiates anti-PD-1 therapy in intrahepatic cholangiocarcinoma
Source: Front Immunol. 2025 Dec 12;16:1708962. doi: 10.3389/fimmu.2025.1708962 (PMC12740922; doi:10.3389/fimmu.2025.1708962)
Supplement: Supplementary file 1 [file DataSheet1.docx]

Supplementary Material

# Supplementary Figures


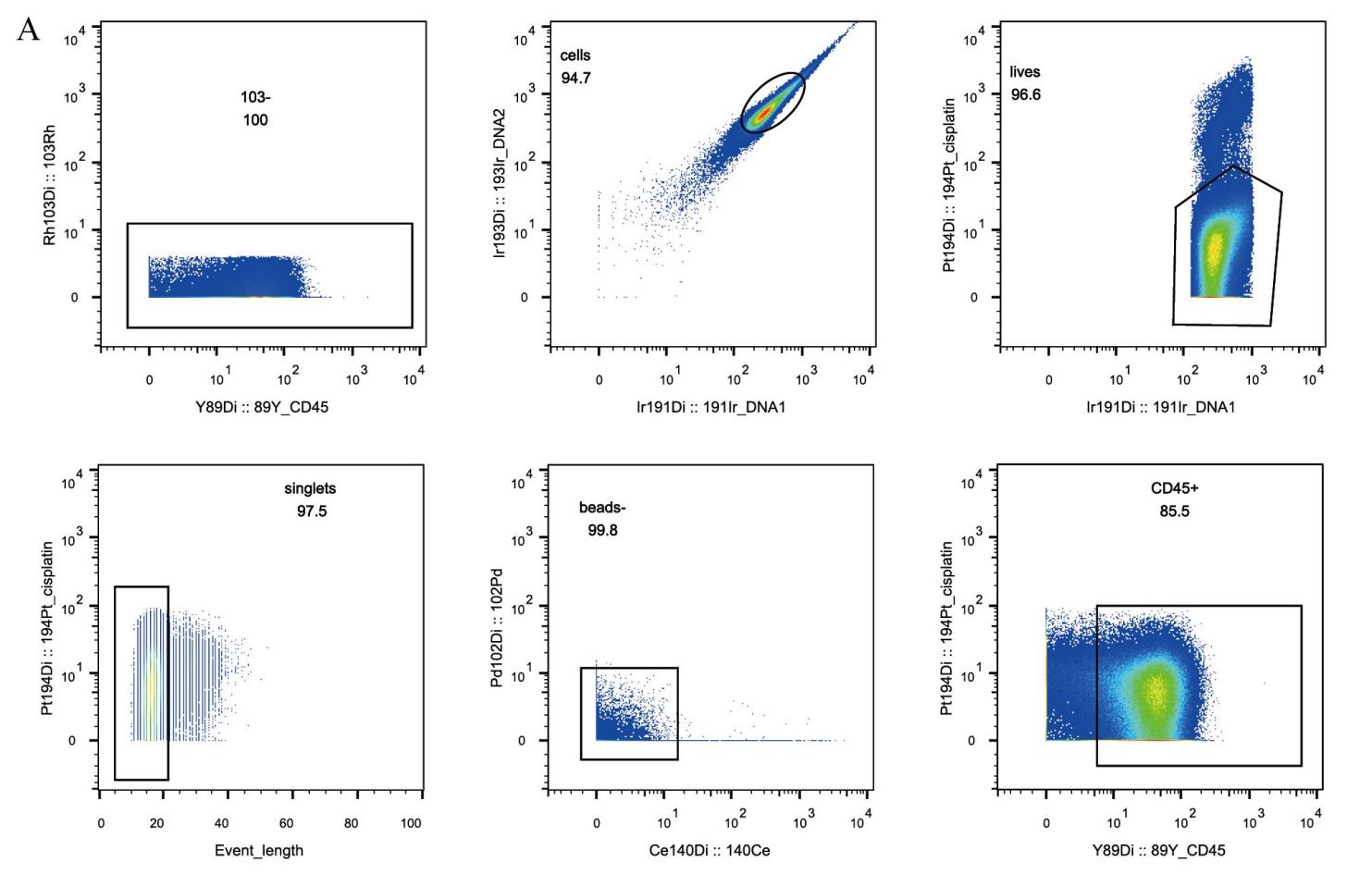


**Supplementary Figure 1.** **CyTOF analysis of tumor-infiltrating immune cells in AKT/YAP murine iCCAs.** (A) Gating strategy to identify live, single, and CD45^+^ immune cells in AKT/YAP murine iCCAs.

**
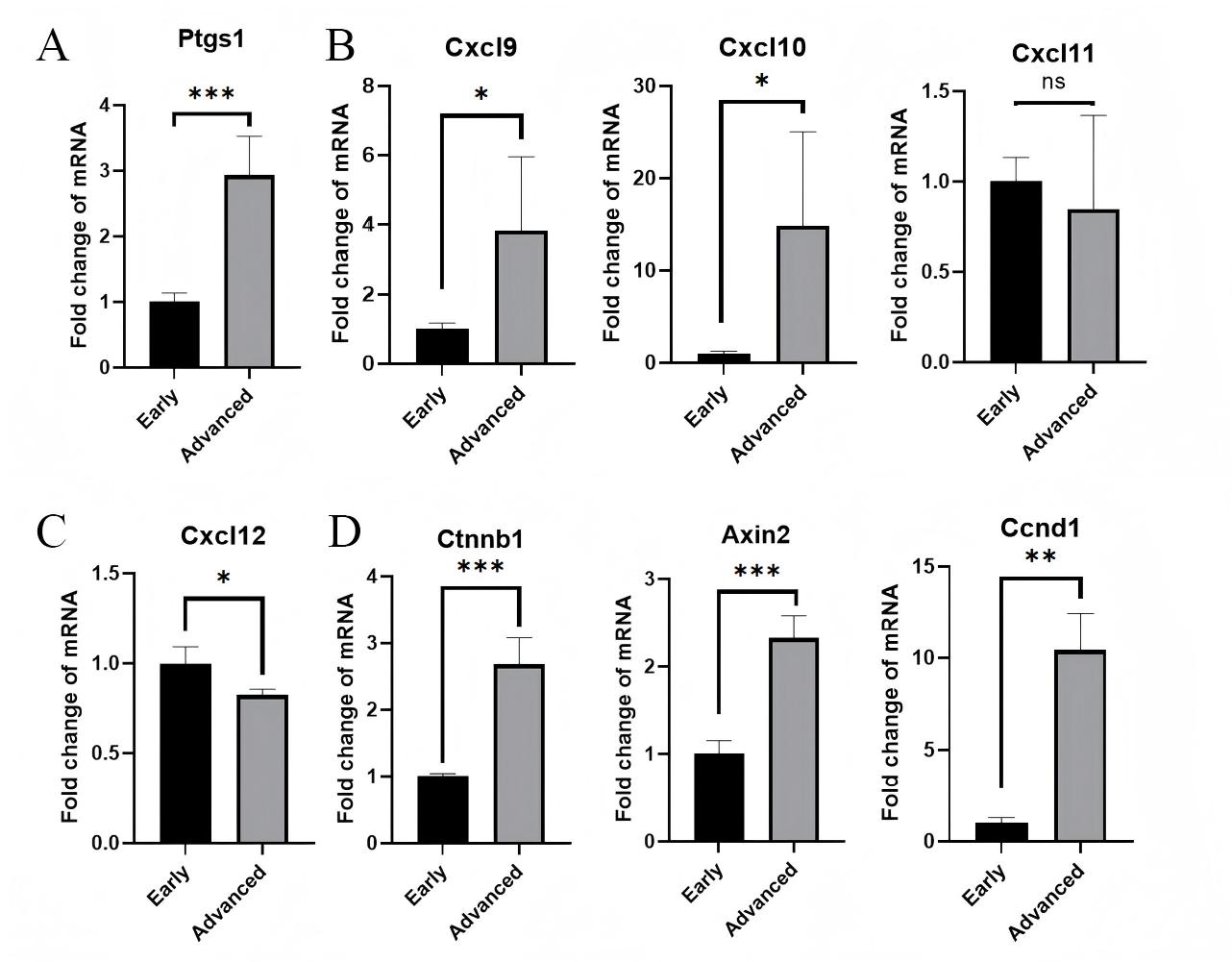
**

**Supplementary Figure 2. Evaluating the expression levels of several tumor-derived factors known to impact cDC1 recruitment/function by performing qPCR of frozen mouse iCCA tissues collected from early and advanced stages (n=4).** Fold change of mRNA levels of PTGS1 （A), CXCL9/10/11/12（B-C), and β-catenin target genes (D) in early and advanced murine iCCAs.

**
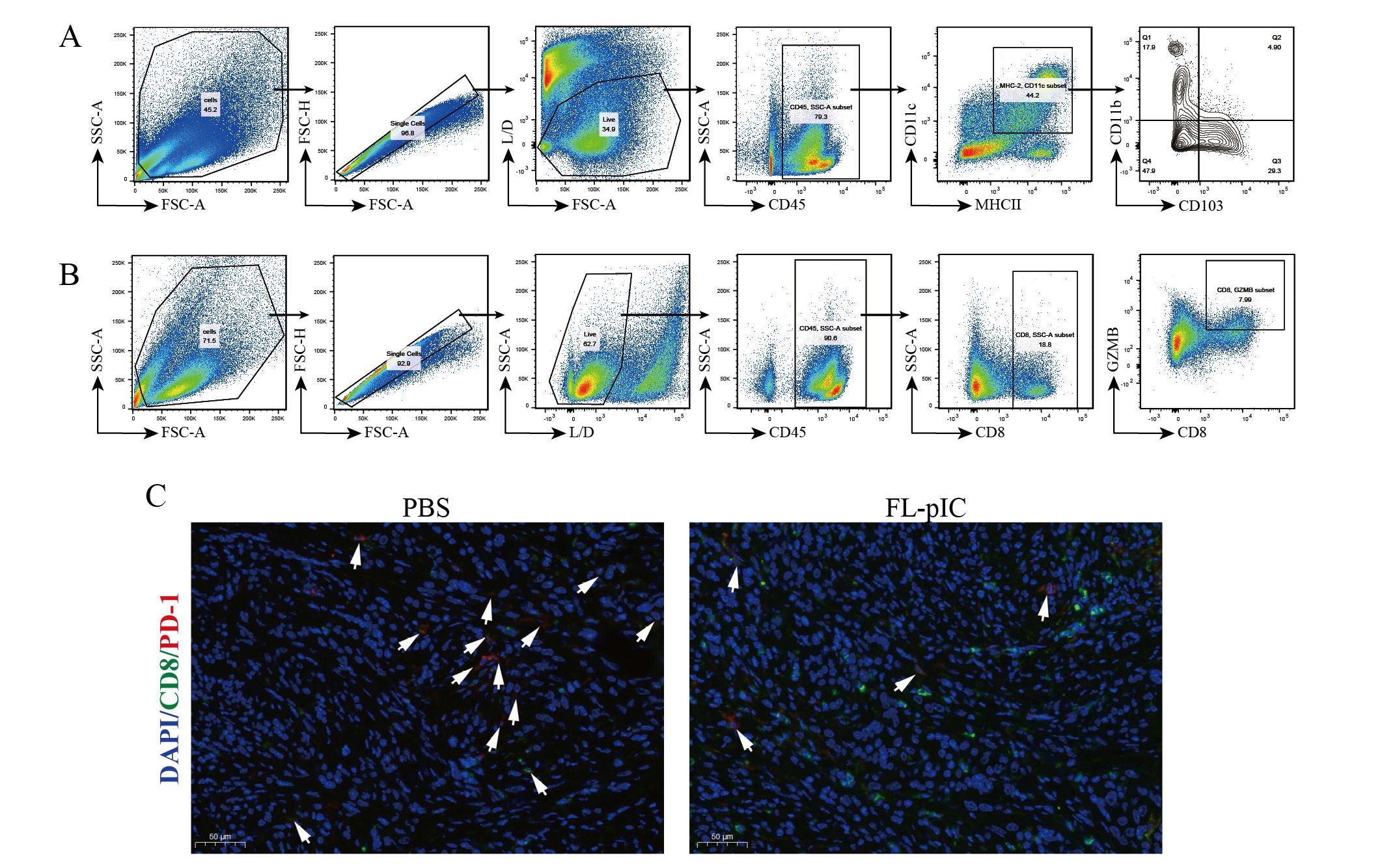
**

**Supplementary Figure 3. Analysis of tumor-infiltrating DCs and CD8^+^ T cells in AKT/YAP murine iCCAs.** Gating strategy to identify intra-tumoral DCs, cDC1s, and cDC2s (A), along with CD8^+^ T cells and CD8^+^ GZMB^+^ T cells (B). Numbers represent % cells within depicted gate. (C) Representative immunofluorescence images of CD8⁺ PD-1^+^ T cells (400X, scale bar, 50μm). White arrows indicate CD8⁺ PD-1^+^ T cells.


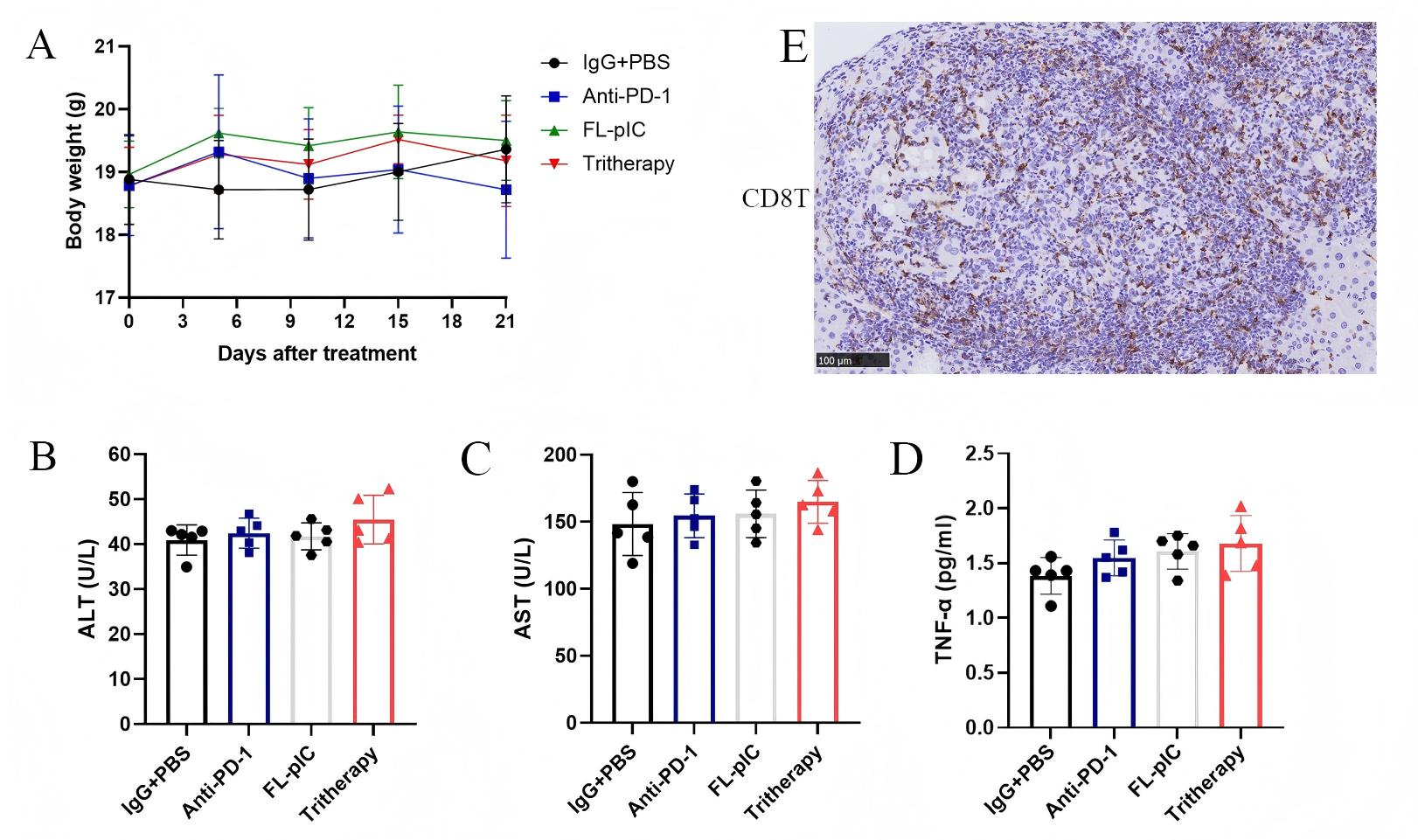
**Supplementary Figure 4. Effects and tolerability of treatment regimens on mouse health.** (A) Body weight curves of mice from indicated groups after treatment. Liver function tests including ALT (B) and AST (C), as well as serum inflammatory cytokine TNF-α (D) level of mice from indicated groups. (E) Mouse liver sections from an acute liver injury model as a positive control for CD8 staining.
